# Analysis of the Application Value of Ultrasound Imaging Diagnosis in the Clinical Staging of Thyroid Cancer

**DOI:** 10.1155/2022/8030262

**Published:** 2022-06-08

**Authors:** Fengying Zhang, Yunxuan Sun, Xijiang Wu, Chunrong Meng, Meiling Xiang, Tingting Huang, Wenping Duan, Fangfang Wang, Zhaolan Sun

**Affiliations:** ^1^Otorhinolaryngology, Weifang Hospital of Traditional Chinese, Weifang, 261000 Shandong, China; ^2^General Surgery, Weifang Hospital of Traditional Chinese, Weifang, 261000 Shandong, China; ^3^Ultrasonography Department, Weifang Hospital of Traditional Chinese, Weifang, 261000 Shandong, China; ^4^First Clinical Medical College, Shandong University of Traditional Chinese Medicine, Jinan, 250000 Shandong, China

## Abstract

Thyroid cancer affects 1.3 percent of the population, with rates of occurrence rising in recent years (approximately 2 percent per year). Thyroid cancer is a common endocrine cancer with an annual increase in occurrence. Although the general prognosis for differentiated subtypes is favorable, the rate of mortality linked with thyroid cancer has been steadily progressing. The presence of suspicious thyroid nodules necessitates more diagnostic testing, including laboratory evaluation, additional imaging, and biopsy. For clinical staging and appropriate patient therapy design, accurate diagnosis is necessary. In this paper, we examined the application value of ultrasound imaging diagnosis in the clinical staging of thyroid tumor in this research. The benefit of early diagnosis is determined in this article using ultrasonography reports from Chinese patients. Images of benign and malignant thyroid nodules were collected and annotated in this work, and deep learning-based image recognition and diagnostic system was built utilizing the adaptive wavelet transform-based AdaBoost algorithm (AWT-AA). The system's efficacy in diagnosing thyroid nodules was assessed, and the use of ultrasound imaging in clinical practice was studied. The variables that had a significant impact on malignant nodules were studied using logistic multiple regression analysis. The sensitivity and specificity of ultrasonography thyroid imaging reporting and data system (TI-RADS) categorization outcomes for benign and malignant tumors were also calculated.

## 1. Introduction

Improved diagnostic imaging and surveillance have led to an increase in thyroid cancer cases worldwide. The sixth most common malignancy is thyroid carcinoma. A patient with this ailment is almost certain to be encountered by almost any healthcare professional during their career. In order to avoid overtreating individuals with lower-risk illness or benign thyroid nodules, the physicians' treatment of thyroid cancers must balance therapeutic methods. Simultaneously, they must identify individuals with developed or higher-risk disease who require a more rigorous therapeutic approach. In the thyroid gland, tumors may range from slow to invasive, with a moderate death rate [1].

Thyroid cancers have surged by 2.4 times in the previous 30 years, according to research. This is one of the greatest increases in any type of cancer that has ever occurred. Thyroid cancer is more likely to occur as people get older. Thyroid cancer strikes females twice as often as it strikes men. However, once impacted, men have a worse prognosis than women. Thyroid cancer usually appears as the painless nodules in the neck's thyroid area. Other symptoms include increased lymph nodes, soreness in the front of the neck, and a variation in voice owing to recurrent laryngeal nerve involvement. Thyroid nodule diagnostic tests divide nodules into two groups: benign and malignant. In this instance, surgical therapy of the nodules is not indicated until the lesion enlarges or creates problems [2].

Thyroid nodule evaluation requires the use of ultrasonography. Ultrasonography has been widely employed in a variety of functions, involving fine-needle aspiration (FNA) biopsy guidance, postoperative assessment, nodule identification, and diagnosis, because of its lower cost and higher sensitivity. However, radiologists typically make diagnoses depending on the sonographic properties of nodules in ultrasound pictures that is subjective and heavily reliant on the radiologists' clinical experiences [3]. Ultrasonography performed in a clinic or office can image thyroid nodules in real time and provide additional information that might otherwise be missed by a history and physical assessment or a partial referral ultrasound. The advantage of thyroid ultrasound over physical assessment is that it permits the assessment and characterization of thyroid nodules, the whole thyroid gland, and the cervical lymph nodes that may vary treatment in more than 60% of patients with a solitary thyroid nodule [4].

In this work, the adaptive wavelet transform-based AdaBoost algorithm is used to analyze the clinical usefulness of ultrasound imaging diagnostics in the clinical stage of thyroid cancer. Section II, which includes the related works, is where the extraneous portions of this investigation are collected. Section III explains the procedure. Section IV examines and assesses the methodology's effectiveness. Section V concludes by providing a concise summary of the findings.

## 2. Related Works

In [5], using the YOLOv2 neural network and deep learning, the author developed an automatic picture recognition and diagnostic method for benign and malignant thyroid nodules. For this study, artificial intelligence was examined for its ability to diagnose thyroid nodules, and its utility was assessed.

In [6], the author mentions that there are around 5% of women and 1% of males who suffer from thyroid nodules. Ultrasonography can detect up to 76 percent of thyroid nodules that are invisible to the naked eye. A deep convolutional neural network (CNN) framework for cytological evaluation of thyroid nodules was the goal of this study.

In [7], the author explains about the more aggressive form of thyroid cancer, medullary thyroid carcinoma (MTC). Using ultrasound (US), thyroid nodules shall now be more accurately categorized as benign or malignant, depending on whether or not ultrasound indications of malignancy are present. Ultrasonography features and MTC biological behavior were examined as part of the study's goal.

In [8], the author discusses the imaging modalities and their part in thyroid tumor detection and maintenance. To that aim, we plan to spotlight cutting-edge diagnostic methods for thyroid cancer in order to increase diagnostic specificity and accuracy.

It is mentioned in [9] that an analysis of incidence and mortality details for females and males in the United States for major thyroid tumor histologic kinds and tumor sizes was conducted in this study to analyze the prevalence rates of subclinical tumor in entire thyroid gland autopsies in people with no known diseases.

In [3], the author explains that about an increasing number of people over 65 are diagnosed with thyroid cancer each year. The study's objective is to examine the long-term relationship between the usage of area-level imaging and the occurrence of thyroid cancer. Thyroid cancer is more likely to be discovered in patients living in places where imaging, particularly thyroid ultrasonography, is more common. They looked into this problem by utilizing the vast variances in imaging between different regions and different points in time. The researchers also expected to be able to identify subgroups of people at risk for thyroid cancer diagnosis utilizing ultrasonography as a first imaging method using Medicare data.

For the purpose of providing an evidence-based approach for treating these tumors, highlighting the lack of proof behind guideline recommendations, and identifying variations in diagnosis and therapy throughout recent decades, the author of [10] conducts a review on follicular thyroid cancer. To determine malignancy from cytological material, ultrasonographic features may be deceiving. Follicular thyroid tumor and Hürthle cell carcinoma differ greatly in presentation and prognosis, yet there are no specific recommendations in evidence-based guidelines.

In [11], the author mentions that a fine-needle aspiration biopsy can only be conducted under ultrasound guidance if necessary to rule out thyroid cancer, according to the author. A diagnosis is made based on the patient's clinical, ultrasonographic, and cytological information.

In [12], the author explains the difficulty to detect nodules with ultrasonic imaging. Currently, radiologists perform this activity manually in clinical practice, which is time-consuming, subjective, and significantly dependent on their clinical expertise. They proposed new deep neural network framework with well-planned loss function regularization and network hyper parameters to detect nodules without the need for expensive post-processing refinement processes. The proposed approach is dependent on the multitask framework Mask R-CNN and uses a deep learning framework to train. In order to avoid unnecessary segmentation, they developed a regularized loss function.

In [13], the author explains that thyroid tumor is the most frequent endocrine tumor in humans, and it is elevating in prevalence. It is difficult to distinguish thyroid tumor from benign thyroid nodules, which occurs in 50% of the population over the age of 50; and it is difficult to stage thyroid cancer to allow for appropriate aggressive surgery in a single session. The study's purpose is to lay forth the fundamentals of good multimodal imaging for thyroid cancer and to help doctors avoid common errors.

In [14], the author explains the purpose of evaluating the diagnostic utility of the TI-RADS; the author cites the TI-RADS classification and pathological data [14] in his study. Patients with thyroid nodules can use the TI-RADS score to determine whether or not more invasive testing is necessary for a proper diagnosis and treatment.

In [15], the author explains that lymphatic drainage channels and enhancement patterns were employed to determine the sentinel lymph nodes and the targeted lymph nodes. Each targeted node was provided a score and recommended for ultrasound-guided fine-needle aspiration depending on the features of conventional US and enhanced patterns. US lymphatic imaging was used in this study as a way to determine the involvement of central lymph nodes in PTC patients prior to surgery and the method's potential for assessing nodal burden, which could offer objective data to help guide surgical decisions.

In [16], the author explains that the thyroid nodule identification, segmentation, classification, and feature extraction methods are shown by providing and discussing noteworthy trends. The benefits and drawbacks of various CAD methods are discussed in detail.

In [17], the author explains about that the contrast-enhanced computed tomography (CECT) is increasingly being used in thyroid tumor individuals, while ultrasonography is the gold standard for assessing cervical lymph node metastases (CT). In order to assess the diagnostic behavior of CT in the identification of metastatic cervical lymph nodes and to determine the factors that lead to diagnostic heterogeneity, they performed a systematic survey and meta-analysis of the literature.

In [18], the author mentions that thyroid cancer incidence estimates and treatment can be affected by the method of thyroid cancer detection used. In order to find out how common it is for tumors to be identified accidentally and to pinpoint the factors that lead to an accidental thyroid cancer diagnosis, the researchers combed through a large number of literature describing thyroid cancer detection methods.

In [19], using task-specific prior knowledge, the author proposes a novel CAD system based on deep learning for autonomous nodule recognition and categorization in ultrasound images in [19]. It is proposed that their CAD system has two steps. A first multiscale detection network is built to understand the features of pyramids for spotting nodules at various scales. The region proposals are constrained by our prior understanding of the size and shape dispersions of actual nodules. Then, a multibranch categorization network is built, wherein every branch collects and improves a particular category of features that radiologists typically use.

In [20], the author implies that when it comes to subjective diagnosis concerns, which are typically dependent on a patient's personal experience, computer-aided diagnostic (CAD) technology can help. A CAD system is made to identify between malignant and benign thyroid nodules on ultrasound images depending on deep learning methodologies. Radiologists' diagnostic abilities were contrasted to those of the CAD system.

In [21], the author explains that malignant nodules can be detected by using an MRI-based “computer assisted diagnostic” (CAD) system. A multi-input convolutional neural network is used in our method to merge the diffusion weighted image (DWI) and “apparent diffusion coefficient” (ADC) maps from MRI scans. There are a number of key advantages to their system. Convolutional neural networks (CNN) can be used to classify thyroid DWI and ADC images, increasing the probability of identifying deep texture structures in thyroid nodules. Additionally, additional channels can be added to each input, allowing for integration with other MRI modalities and imaging technologies such as diffusion tensor imaging (DTI).

Earlier, investigations have reported inconsistent outcomes when it comes to the diagnostic efficiency of CT in the assessment of lymph node metastases, and the utility of CT stays unknown, according to [22]. As a result, the author's purpose was to see if combining CT with ultrasound could aid in the identification of lymph node metastases in thyroid tumor individuals.

In [23], the author mentions that 40 thyroid nodules were studied and that many variables, such as histogram parameters and fractal dimensions, were retrieved from the data. Support vector machines and random forests classifiers were used to categorize nodules into malignant and benign groups based on the attributes.

In [24], the author explains that assessing cervical lymph node metastasis (LNM) in patients with papillary thyroid cancer (PTC), ultrasound is the first imaging modality that is used. Even in those with LNM, the computer tomography (CT) has an impact on the surgical procedure. Ultrasound and computed tomography have been found to be beneficial in identifying cervical LNM.

## 3. Proposed Work

**Figure 1:**
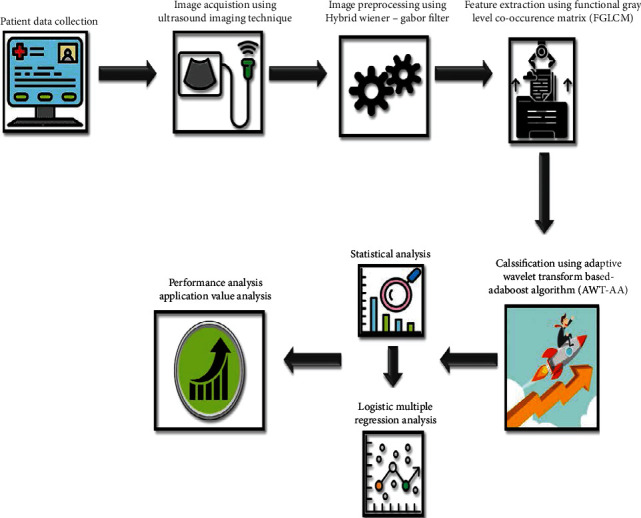
Flow of the illustrated work.

Our proposed work focused on designing an automatic diagnosis system for differentiating thyroid nodules into benign and malignant nodules for diagnosing the clinical stages of thyroid cancer based on ultrasound images. The approach involved preprocessing, feature extraction, and classification. Initially, the acquired images were preprocessed using a hybrid Wiener–Gabor filter. Then, important features were extracted using the functional gray level Cc-occurrence matrix (FGLCM). Finally, the thyroid nodules were classified using AWT-AA model based on the extracted features. The detailed flow of our work is illustrated in Figure 1 and explained in this section.

### 3.1. Patient Data Collection and Image Acquisition

Between Jan 2016 and Oct 2020, 384 cases suffering solid thyroid nodules were documented at the Weifang Hospital of Traditional Chinese. Individuals' detailed health information, including essential patient records, medical diagnosis, lab test results, inspection analyses, and clinical notes, among other things, was gathered. They never should have had any previous medical interventions. The ultrasound pictures of their thyroid nodules have been obtained using ultrasonography. The ultrasound equipment (GE Logiq E9, S7) was used to capture all of the pictures, with probe frequency set to 5-12 mHz or 8-15 mHz. The database contains 450 thyroid nodule photos from 384 individuals, 322 of whom are cancerous and 128 of whom are harmless. Fine needle aspiration (FNA) biopsies and pathology findings were used to determine if a nodule is cancerous or normal.

### 3.2. Image Preprocessing Using Hybrid Wiener-Gabor Filter (HWGF)

During the capture of the image data, distortion and extraneous indications could well have happened. As a result, pictures should be standardized before beginning subsequent image analytical procedures in order to reduce noise and improve image quality. For preprocessing, the images were acquired in this investigation; we can use a HWGF. This method is a combination of the Wiener and Gabor filters. The pictures are first treated with a Wiener filter to eliminate Gaussian noise. The pictures are then run through a Gabor filter to improve the texture clarity of the pictures even more.

The Wiener filter is preferred because it is straightforward, quick, and also has a reduced noise-related peak signal. This is usually applied in the deconvolution approach via linear invariant technique to diminish distortion. Equation (1) gives the kernel's convolution with the picture's pixels, which is specified by a Gaussian function:

(1) $$\begin{array}{c} c*g\left[v\right]≝\overset{\infty }{\underset{l=-\infty }{\sum }}c\left[l\right].g\left[v.l\right]. \end{array}$$

Equation (2) defines the 2-dimensional Gaussian function [c(f,g)], which is employed to construct the kernel with size (3 × 3).

(2) $$\begin{array}{c} c\left(f,g\right)=A.e^{-\left(\frac{{\left(f-f_{0}\right)}^{2}}{2\sigma f^{2}}+\frac{{\left(f-g_{0}\right)}^{2}}{2\sigma g^{2}}\right)}. \end{array}$$

Here, A indicates the magnitude, (f_0_, g_0_) depicts the center, and *σ*f and *σ*g mean the standard deviations in the *x* and *y* directions.

Weiner was deemed authentic since it employs a linear equation method to calculate a collection of ideal filter weights that reduce the noise level of a received data. It analyzes the bend correlation as well as covariance matrices of noisy data in order to determine such weights, and it gives an accurate assessment of an identifiable stochastic message under linear distortion. The distortion values are calculated and employed to determine the ideal weight of the filter. The Wiener filter would be used to remove noise from a recognized distorted picture based on data derived from a local neighborhood of every pixel by assessing a new input data having similar noise attributes with optimal filtering weights. As a result, the input photos are free of Gaussian noise.

To enrich the texture information, the denoised picture acquired from the Weiner filter is processed through the Gabor filter. Corners, edges, and blobs were identified using Gabor kernels. Gabor parameters were calculated by the Fourier transform of an assessed physiological signal in this space-frequency relation. The width and phase of the Fourier transform is used to determine Gabor functions based on the amplitude and phase of an input signal at specified frequencies. The Gabor transform synchronizes with the input to clearly identify image edges. Equation (3) defines the specific formula of the Gabor kernel (*G*_*fg*_).

(3) $$\begin{array}{c} G_{fg}=Ae^{-q\left(Ffcos\theta +Fgsin\theta \right)}e^{-0.5\left(\frac{Z_{1}^{2}}{\sigma_{1}^{2}}+\frac{Z_{2}^{2}}{\sigma_{2}^{2}}\right)}. \end{array}$$

Here, *F* = frequency and *θ* = orientation, respectively, *A* = constant, and *Z*_1_ and *Z*_2_ are sinusoidal functions.

Convolution with the picture is performed using numerous Gabor kernels, referred to as a Gabor bank, in the process of extracting maximum information from different frequencies and orientations. As a result, HWGF is used to augment the input pictures.

### 3.3. Feature Extraction Using Functional Gray Level co-Occurrence Matrix (FGLCM)

Medical pictures contain a wealth of textural data that is important in clinical research. The textural features detected in ultrasound medical imaging of the thyroid gland aid in the differentiation of a malignant or benign thyroid nodule from a normal thyroid nodule. We employed a texture feature extraction technique relying on FGLCM. Equation (4) defines the co-occurrence matrix P for a grayscale picture I of size VxV:

(4) $$\begin{array}{c} P\left(q,z\right)=\overset{V}{\underset{e=1}{\sum }}\overset{V}{\underset{g=1}{\sum }}\left\{\begin{array}{cc} 1; & Q\left(e,g\right)=qandQ\left(e=∆e,g+∆g\right)=z\\ 0; & otherwise \end{array}\right\}. \end{array}$$

The offset specifies the distance between a particular pixel and its next-door neighbors (*Δ*e, *Δ*g). The co-occurrence matrix is susceptible to rotation when the offset (*Δ*e, *Δ*g) parameterization is used. The identical (rotated) images will have a various co-occurrence matrix if the offset vectors are chosen so that the rotations of the images are not equal to 180 degrees. It could be prevented by forming the co-occurrence matrix with a set of offsets sweeping through 180 degrees at the same distance parameter *Δ*, resulting in an extent of rotational invariance of [0 *Δ*] for 0 degree, P horizontal; [-*Δ*, *Δ*] for 45 degrees, P right diagonal; [*Δ*-0] for 90 degrees, P vertical; and [-*Δ* -*Δ*] for 135 degrees, P left diagonal. For the thyroid nodule ultrasound pictures, the FGLCM matrix was constructed. The calculated GLCM matrix was then used to extract texture features. Ten different features extracted for our dataset namely autocorrelation (AC), contrast (CT), correlation (CR), cluster prominence (CP), cluster shade (CS), dissimilarity (D), energy (EN), entropy (ET), homogeneity (H), and maximum probability (MP) are determined:

(5) $$\begin{array}{c} AC=\underset{q}{\sum }\underset{z}{\sum }\left(q,z\right)P\left(q,z\right),\\ CT=\underset{q}{\sum }\underset{z}{\sum }{\left|q-z\right|}^{2}P\left(q,z\right),\\ CR=\underset{q}{\sum }\underset{z}{\sum }\frac{\left(q-\mu e\right).\left(z-\mu g\right)P\left(q,z\right)}{\sigma e.\sigma g},\\ CP=\underset{q}{\sum }\underset{z}{\sum }{\left(q+z-\mu e-\mu g\right)}^{4}P\left(q,z\right),\\ CS=\underset{q}{\sum }\underset{z}{\sum }{\left(q+z-\mu e-\mu g\right)}^{3}P\left(q,z\right),\\ D=\underset{q}{\sum }\underset{z}{\sum }\left|q-z\right|P\left(q,z\right),\\ EN=\underset{q}{\sum }\underset{z}{\sum }{\left|P\left(q,z\right)\right|}^{2},\\ ET=-\underset{q}{\sum }\underset{z}{\sum }P\left(q,z\right)\log\left(P\left(q,z\right)\right),\\ H=\underset{q}{\sum }\underset{z}{\sum }\frac{P\left(q,z\right)}{1+\left|q-z\right|},\\ MP=\max_{q,z}P\left(q,z\right). \end{array}$$

Here, p(q, z) represents the (q, z) gray-tone of GLCM. The retrieved texture aspects are then employed for additional categorization procedure.

### 3.4. Classification of Thyroid Nodules Using Adaptive Wavelet Transform-Based AdaBoost Algorithm (AWT-AA)

The classification of thyroid nodules was carried out depending on the features extracted for ultrasound thyroid nodule images using AWT-AA strategy. The model is designed based on the concepts of wavelet transform and Adaboost algorithm. Other than texture features, adaptive wavelet transform (AWT) features were extracted at the first stage of classification process.

The term AWT refers to the wavelet idea. Wavelets are sophisticated mathematical instruments for data analysis. Mother wavelets that have been scaled and transformed could be used to breakdown an input. It decomposes signals into low and high pass elements using filter banks made up of finite impulse response filters. The low pass element provides data about image properties that change slowly, while the high pass element includes data about image changes that happen quickly. Filtering both rows and columns of images with low pass filters yields coefficients that indicate how much energy is in each image. The resulting coefficients include the vertical features of the image when low pass filtering is applied to the rows and high pass filtering is conducted on the column values. The coefficients are produced through row-wise high pass filtering and column-wise low pass filtering, and they include the image's horizontal features. The finest-scale coefficients are produced via high pass filtering of both row and column values, and they contain the image's diagonal features. These details are also taken into consideration along with texture features for classification process.

Then, the AdaBoost algorithm is applied for classifying the images. It is a machine learning algorithm. It is adaptive since the examples miscategorized by the first classifier are restructured into the succeeding classifiers to increase classification performance, and it is frequently utilized to improve the performance of several other poor learning algorithms. The enhancing approach gives all examples in the training data equal weights at first. The weight is redistributed for each example based on the output of classifiers on this training set. When reallocating the weights, the one for each correctly identified example is reduced, whereas the one for incorrectly classed instances is enhanced. A classifier reformulates on this reweighted data in the next round, attempting to accurately identify the examples with increased weight. The weights are adjusted once more based on the output of the new classifiers. When the weights are adjusted, the normalization is done to keep the sum of the weights the same as it was before. The final hypothesis value is obtained after all rounds. The final hypothesis is either 0 or 1, which is what the strong classifier H(a′) in Equation (6) predicts.

(6) $$\begin{array}{c} H\left(a^{\prime }\right)=\left\{\begin{array}{cc} 1 & \text{if}\overset{T}{\underset{t=1}{\sum }}\log\frac{1}{\propto_{t}}H_{t}\left(a^{\prime }\right)\geq \frac{1}{2}\overset{T}{\underset{t=1}{\sum }}\log\frac{1}{\propto_{t}}\\ 0 & otherwise \end{array}\right. \end{array}$$

The strong classifier constructed using AWT-AA is used for classifying the thyroid nodules into benign and malignant nodules.

### 3.5. Statistical Analysis Using Logistic Multiple Regression Algorithm

The researchers looked at the link between patients' essential principles and benign and malignant thyroid cancers. The risk variables for thyroid hormone in thyroid nodules were studied using logistic multiple regression analysis. The influence of categorization on the diagnosis of thyroid nodules was investigated using the TI-RADS classification system for ultrasonography diagnostics. Sensitivity, specificity, and accuracy values were all computed. The area of the receiver operating characteristic (ROC) curve was constructed to examine the link between ultrasound findings and the final benign or malignant prognosis.

## 4. Result and Discussion

The Matlab simulation tool is used to test the proposed approach. Specifications such as (a) accuracy, (b) specificity, (c) sensitivity, and (d) area under curve (AUC) are used to validate the suggested approach's behavior. This evaluation will take into account four factors: *t*_*p*_ indicates true positive, *t*_*n*_ indicates true negative, *f*_*p*_ indicates false positive, and *f*_*n*_ indicates false negative.

- *t*_*p*_denotes that the data is normal, and it turned out to be exactly that
- *t*_*n*_ denotes that the data is expected to be affected by tumor, and it is really affected
- *f*_*p*_ denotes that the data is expected to be affected by tumor, yet it is a normal data
- *f*_*n*_ denotes that the data is expected to appear normal data, however it is an affected one

Here, we compare the proposed adaptive wavelet transform-based AdaBoost (AWT-AA) with the existing methods such as clinical knowledge guided multiscale detection network (CKG-MDN) and SVM-based computer-aided diagnosis.

**Figure 2:**
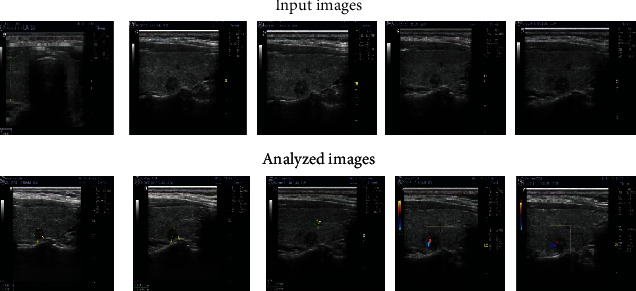
Samples of input images and analyzed images observed during ultrasound diagnosis of thyroid nodules.

Figure 2 depicts the enhancement and high-lighting of the input ultrasound images for the assessment procedure. Thyroid nodules are blurred in the input pictures. The clear thyroid nodules were displayed in the analyzed pictures through the highlightened regions.

### 4.1. Accuracy

It determines the number of data that are successfully classified. It decides how closely the outcomes match the initial outcome. To get an idea of a test's accuracy, we should look at the percentage of true positive and false negative results across all cases:

(7) $$\begin{array}{c} Accuracy=\frac{t_{p}+t_{n}}{t_{p}+t_{n}+f_{p}+f_{n}}. \end{array}$$

**Figure 3:**
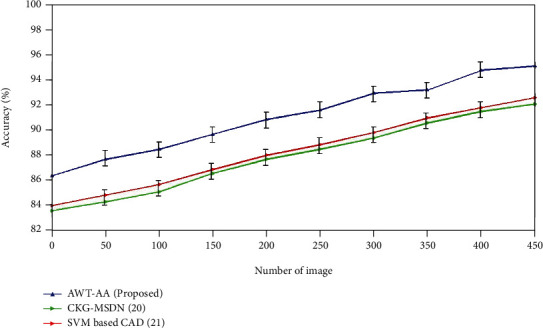
Comparative analysis of accuracy with existing and proposed method.

Figure 3 depicts the accuracy ratings for the currently employed and newly proposed approaches. The SVM-based CAD method provides the accuracy of 92.5% which is higher when compared to the accuracy of CKG-MDN which is 92%. The proposed AWT-AA acquires the accuracy of 95% which is far better than the existing methods.

### 4.2. Sensitivity

A test's sensitivity refers to its capacity to appropriately identify patient cases measures how many people test positive for the disease out of all the persons with the ailment. Compute the fraction of true positives in patient instances to evaluate it. It could be defined quantitatively as

(8) $$\begin{array}{c} \text{Sensitivity}=\frac{{\text{t}}_{\text{p}}}{{\text{t}}_{\text{p}}+{\text{f}}_{\text{n}}}=\frac{t_{p}}{\text{total number of ill person in the community}}=\text{probabilities that the test will be positive if the person has the disease}. \end{array}$$

**Figure 4:**
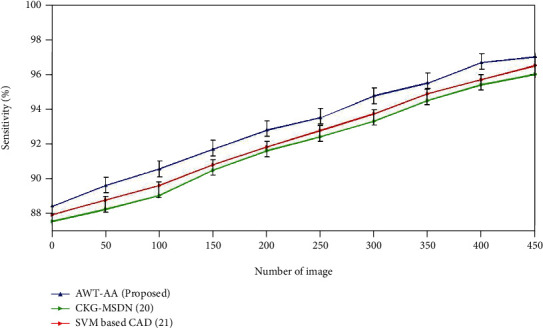
Comparative analysis of sensitivity with existing and proposed method.

In order to rule out a disease, it is important to get an accurate test result that does not show anything. People who have the disease are rarely misdiagnosed by tests that have a high sensitivity. A test with 100% sensitivity will be able to tell if a person has the disease if they test positive. A negative test result would be a sure way to say that a patient does not have the disease. However, a positive result in a test with a high level of sensitivity does not always mean that you have a disease.  Figure 4 depicts the sensitivity ratings for the currently employed and newly proposed approaches. The SVM-based CAD method provides the sensitivity of 96.4% which is higher when compared to the accuracy of CKG-MDN which is 96%. The proposed AWT-AA acquires the sensitivity of 97% which is superior than the existing methods.

### 4.3. Specificity

The capacity of a test to correctly reject healthy patients without a problem is referred to as its “specificity.” Test specificity is the percentage of people who actually do not have the condition that test negative for the condition. Compute the fraction of true negatives in healthy instances to evaluate it. It could be defined quantitatively as

(9) $$\begin{array}{c} \text{Specificity}=\frac{{\text{t}}_{\text{n}}}{{\text{t}}_{\text{n}}+{\text{f}}_{\text{p}}}=\frac{{\text{t}}_{\text{n}}}{\text{total number of well person in the community}}=probabilities\;that\;\text{the }test\;will\;be\;negative\;\text{if }\text{the }person\;\text{is }well. \end{array}$$

**Figure 5:**
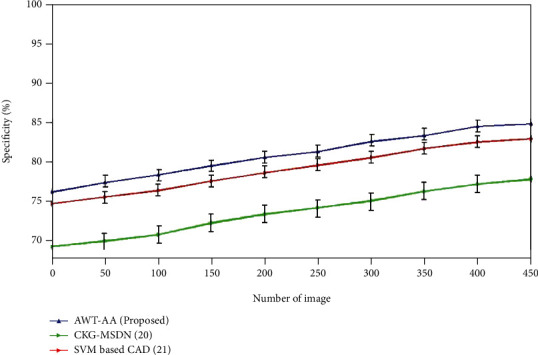
Comparative analysis of Specificity with existing and proposed method.

Figure 5 depicts the specificity ratings for the currently employed and newly proposed approaches. SVM-based CAD method provides the specificity of 83.1% which is higher when compared to the specificity of CKG-MDN which is 78%. The proposed AWT-AA acquires the specificity of 85% which is superior than the existing methods.

### 4.4. Area Under Curve (AUC)

**Figure 6:**
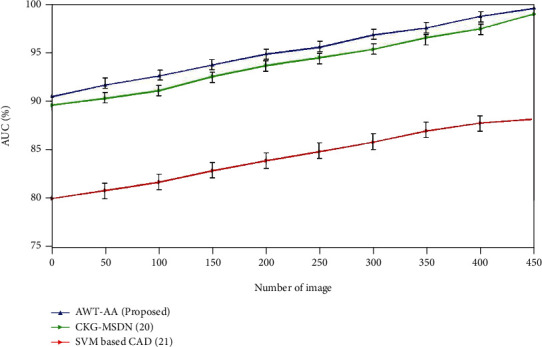
Comparative analysis of AUC with conventional and traditional method.

The shorthand for “area under the ROC curve” is “AUC.” It is a measure of the overall two-dimensional area beneath the ROC curve. AUC is a compound performance statistic that considers all potential classification degrees. One way to look at AUC is to see how likely it is that the system will rate a randomized positive case better than a randomized negative one. AUC comparison is given in Figure 6. AUC percentage of AWT-AA method was higher than the existing methods.

From the above result analyses, it is confirmed that the proposed AWT-AA model exhibited higher performance efficiency in classifying thyroid nodules compared to other existing models. Further, we contrasted the classification performance of our model with the manual classification outcomes produced by the radiologists to analyze the application value of ultrasound imaging diagnosis using AWT-AA in Table 1. The proposed method shows greater accuracy, sensitivity, and specificity compared to the performance of radiologists.

## 5. Conclusion

Thyroid cancer is among the most dangerous cancers that people can have. As a result, it must be diagnosed correctly. Based on ultrasound scans, we utilized the adaptive wavelet transform-based AdaBoost algorithm (AWT-AA) classification model to detect and classify thyroid nodules. Our suggested approach outperformed existing thyroid nodule classification algorithms with the accuracy of 95%. Furthermore, our proposed system outperformed expert radiologists in terms of accuracy, sensitivity, and specificity. As a result, our suggested diagnostic model may effectively identify benign from malignant thyroid nodules. In the future, further enhancements must be carried out to improve the efficiency of classification performance.

## Figures and Tables

**Table 1:** Comparative analysis of existing and contemporary methods.

Method	Accuracy (%)	Sensitivity (%)	Specificity (%)
Radiologists	84	89	71
AWT-AA (proposed)	95	97.5	86

## Data Availability

The datasets used and/or analyzed during the present study can be available from the corresponding author if needed.
